# Utilizing Point-of-Care Ultrasound in the Diagnosis and Management of a Giant Hip Lipoma: A Case Report

**DOI:** 10.7759/cureus.81461

**Published:** 2025-03-30

**Authors:** Grant M Pham, Roger N Pham

**Affiliations:** 1 Family Medicine, Texas Tech University Health Sciences Center El Paso Paul L. Foster School of Medicine, El Paso, USA; 2 Emergency Medicine, Baylor College of Medicine, Houston, USA

**Keywords:** case report, giant lipoma, lipoma, point-of-care ultrasound (pocus), radiation safety, resource limited, surgical excision

## Abstract

Lipomas are common benign soft tissue tumors that often require accurate imaging for diagnosis and management. Point-of-care ultrasound (POCUS) has emerged as a valuable diagnostic tool, providing real-time imaging at the bedside and reducing the need for advanced imaging modalities. We report a case of a 43-year-old male presenting with a large lipoma on his right hip. The lipoma measured 12.9 cm × 14 cm × 12 cm and exhibited a circumscribed, isohypoechoic mass relative to adjacent fat in the subcutaneous layer with no vascularity on POCUS. Utilizing POCUS, we accurately localized the lipoma, avoiding critical nerve and vascular structures. This facilitated a safe, outpatient surgical excision without the need for additional imaging such as MRI or CT scans. The procedure was completed successfully without complications, resulting in significant symptom relief and no recurrence at six months post-operation. The use of POCUS in this case demonstrated its effectiveness in providing immediate, accurate diagnostic information, enabling prompt and cost-effective treatment. In settings where advanced imaging modalities are not readily available, POCUS helps avoid radiation exposure and reduce healthcare costs. POCUS ensured precise surgical planning, enhancing patient safety by preventing damage to surrounding critical structures. In this case, it was effective in distinguishing a benign lesion. However, additional imaging or histopathological confirmation may be necessary in cases with atypical features or diagnostic uncertainty. This case underscores the potential of POCUS as a first-line diagnostic tool in the evaluation of large soft tissue masses, particularly in settings with limited access to advanced imaging or when rapid decision-making is essential. Integrating POCUS into clinical practice can improve workflow efficiency, patient outcomes, and overall healthcare delivery.

## Introduction

Lipomas are the most common benign soft tissue tumors, usually presenting as small, slow-growing, painless subcutaneous masses composed of mature adipocytes [1]. While most lipomas measure less than 5 cm in diameter, giant lipomas, commonly defined in the literature as those exceeding 10 cm, are rare and can present diagnostic challenges [2]. Because of their large dimensions, giant lipomas may clinically mimic malignant soft tissue tumors such as liposarcomas, making accurate imaging and careful evaluation essential to ensure correct diagnosis and management [3].

Imaging studies play a crucial role in the evaluation of soft tissue masses, as misdiagnosing a malignant lesion as benign can lead to delays in treatment, tumor progression, and potentially worsened patient outcomes. MRI is considered the gold standard due to its superior soft tissue contrast and ability to delineate tumor margins [4-5]. However, MRI may not be readily accessible in all settings due to cost and availability. CT scans and radiographs, while informative, involve radiation exposure, which is undesirable when alternatives exist [4-5]. Ultrasound, particularly point-of-care ultrasound (POCUS), offers a valuable alternative for initial assessment [6]. POCUS allows clinicians to perform bedside imaging without radiation exposure, providing immediate diagnostic information that can guide management decisions [6].

We present a clinically significant case of a giant hip lipoma evaluated using POCUS, including Doppler imaging to accurately identify and avoid critical vascular structures. The real-time information from POCUS enabled prompt and safe surgical intervention, highlighting its utility as a cost-effective diagnostic and planning tool, particularly in underserved settings where access to advanced imaging modalities such as MRI or CT is limited or unavailable.

## Case presentation

A 43-year-old male with no significant medical history presented with a progressively enlarging mass on his right hip. He first noticed the mass five years prior, and it had gradually increased in size. The mass caused discomfort during work-related activities, particularly those involving physical exertion such as bending and lifting. These difficulties prompted him to seek medical attention. He reported no bleeding, pain, or pruritus and had no history of trauma or surgery in the affected area. Additionally, he denied experiencing weight loss and fever.

**Figure 1:**
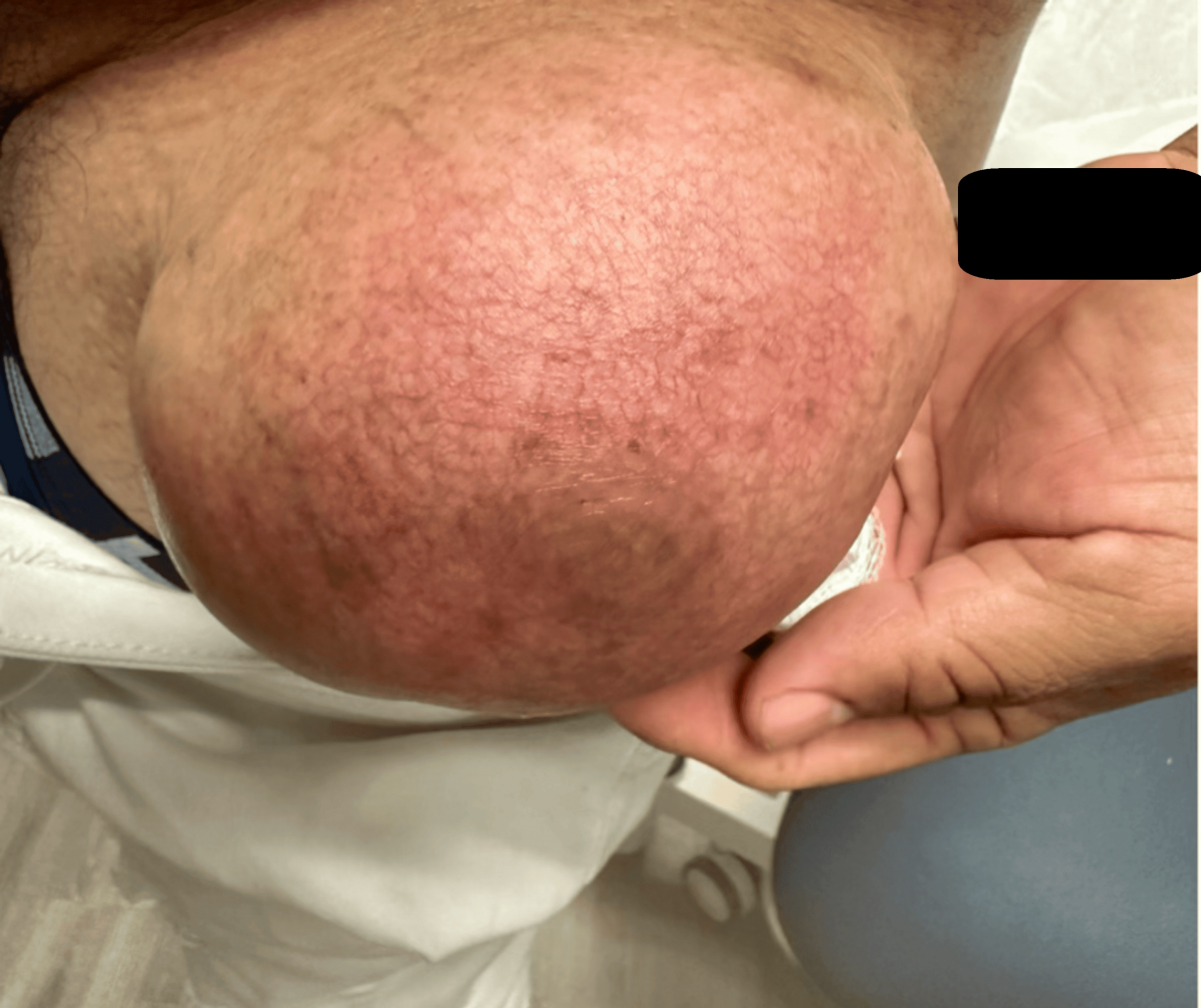
Lipoma on the right lateral hip.

Physical examination revealed a large mass measuring approximately 12.9 cm × 14 cm × 12 cm on the lateral aspect of the right hip. On palpation, the mass was smooth, soft, non-tender, and mobile. The overlying skin appeared normal (Figure 1). There was no evidence of regional lymphadenopathy. Moreover, neurological and vascular examinations of the hip and right lower limb were unremarkable.

**Figure 2:**
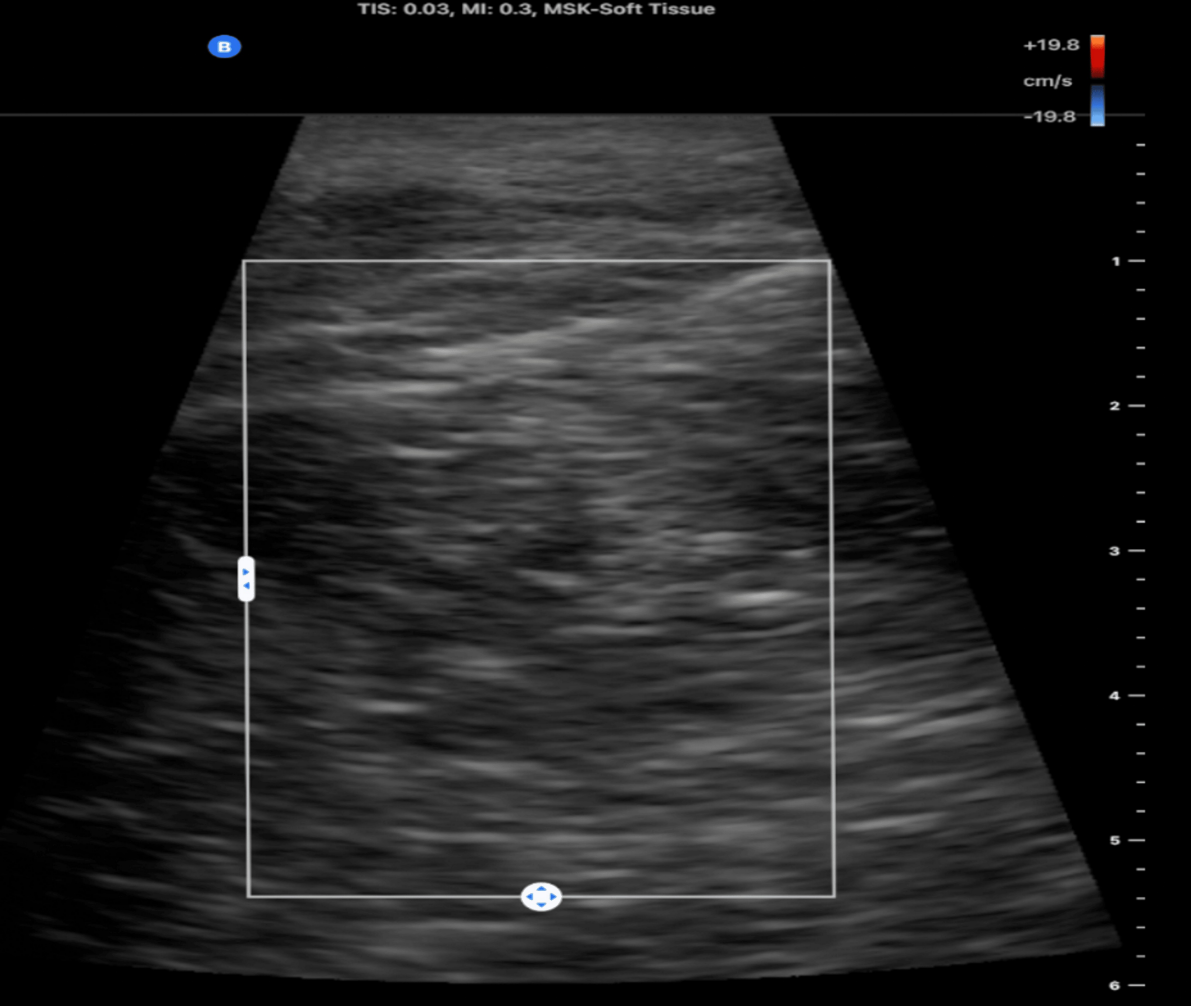
Point-of-care ultrasonographic imaging of the right lateral hip revealed a lipomatous mass.

A POCUS was performed using a high-frequency linear transducer. The ultrasound revealed a circumscribed, homogeneously isoechoic mass in the subcutaneous tissues. Doppler imaging showed no vascularity (Figure 2).

**Figure 3:**
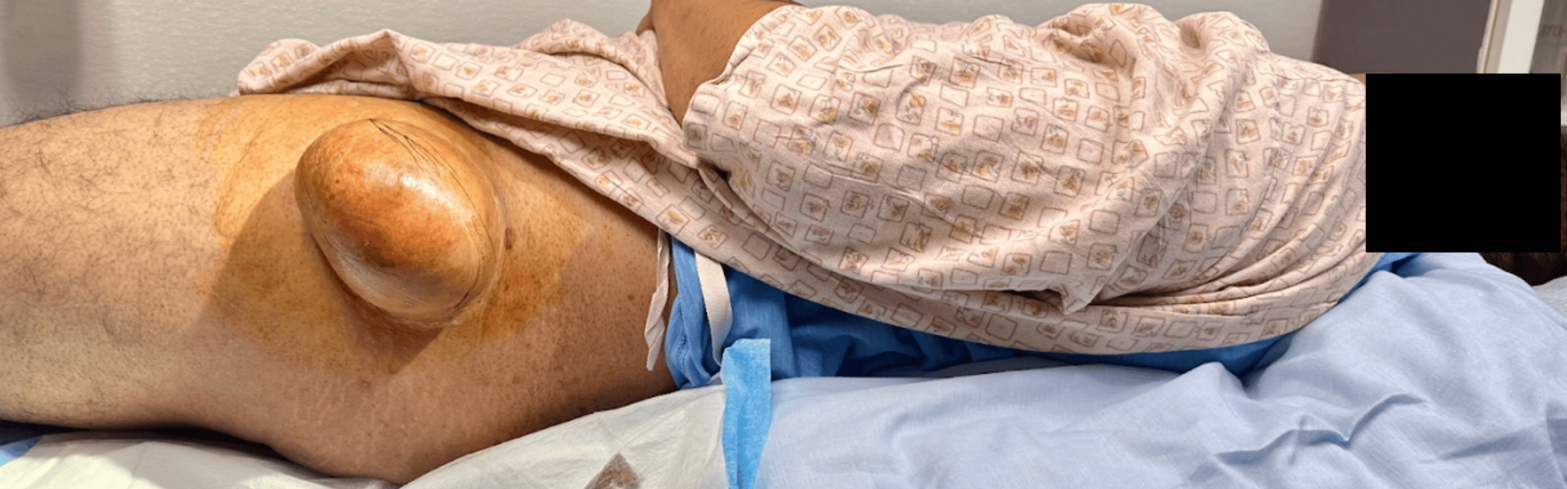
Giant lipoma on the right thigh, being prepped for surgical excision.

**Figure 4:**
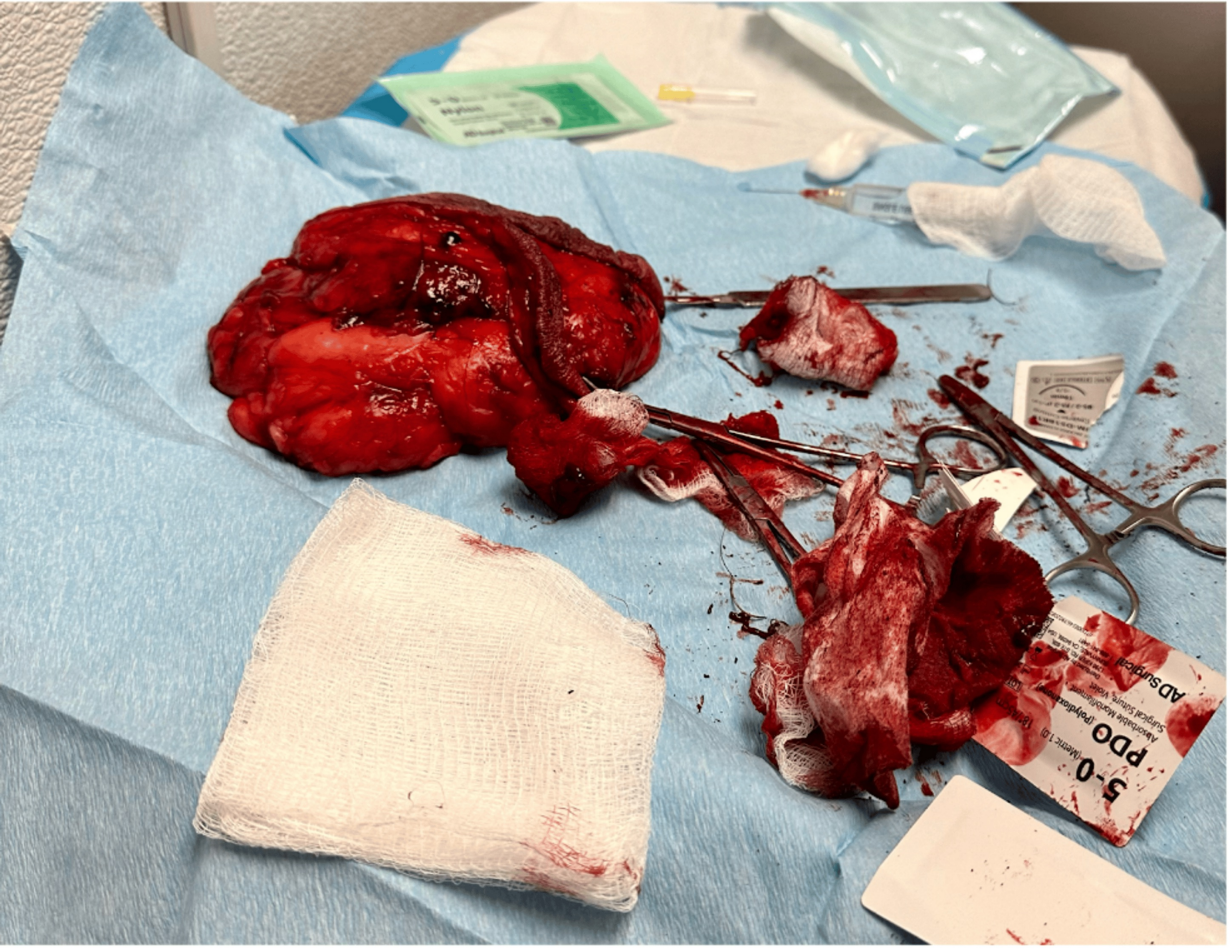
Intraoperative photograph of the excised lipoma measuring 12.9 cm × 14 cm × 12 cm.

These features were consistent with a lipomatous lesion [7]. Removal is indicated for symptomatic lipomas, especially when they are large or causing discomfort [8]. Given the size of the mass and the impact on the patient's daily activities and occupation, surgical excision was performed (Figure 3). The excision was performed on an outpatient basis on the same day as the diagnosis. Under local anesthesia, an elliptical incision was made over the mass. Sharp and blunt dissection techniques were used to separate the mass from surrounding tissues. Two small blood vessels were ligated. The mass was completely excised without complications (Figure 4).

Intraoperative findings showed a well-encapsulated, smooth-bordered mass without infiltration of adjacent structures. The excised specimen measured 12.9 cm × 14 cm × 12 cm and weighed approximately 900 grams.

Histopathological examination confirmed a benign lipoma composed of mature adipose tissue without atypia. The postoperative course was uneventful. At follow-up visits at two weeks, one month, three months, and six months, the patient demonstrated no complications or signs of recurrence. The patient reported significant improvement in comfort during daily activities and work, expressing satisfaction with the cosmetic outcome and relief of discomfort.

## Discussion

Giant lipomas are rare entities that can present diagnostic and therapeutic challenges. Due to their size, they may mimic malignant tumors such as liposarcomas, necessitating careful evaluation [4]. Differentiation between benign and malignant fatty tumors is crucial, as the management and prognosis differ significantly [4].

In this case, the patient's occupation in construction, involving substantial physical labor, exacerbated the functional limitations imposed by the giant lipoma. Activities essential to his job became increasingly difficult, highlighting the impact such tumors can have on quality of life and occupational performance.

POCUS played a pivotal role in the rapid evaluation and management of this patient. As an immediate, non-invasive, and cost-effective imaging modality, POCUS facilitated bedside assessment, providing real-time diagnostic information without radiation exposure. The ultrasound findings of a well-defined, homogeneously hyperechoic or hypoechoic mass without internal vascularity are characteristic of lipomatous lesions [7]. Conversely, liposarcomas commonly present with irregular margins, internal heterogeneity, and increased Doppler vascularity. Although these features can overlap, studies suggest that Doppler imaging findings, specifically the presence or absence of significant internal vascularity, are important sonographic indicators to differentiate benign lipomas from potentially malignant liposarcomas [9-11]. The absence of internal vascularity on color Doppler imaging was reassuring and supported the benign nature of the mass; however, this finding, when used alone, should be interpreted cautiously, as some malignant lesions, such as well-differentiated liposarcomas, can exhibit minimal vascularity [12].

The utilization of POCUS in this case significantly enhanced patient care by providing a confident preliminary diagnosis. This approach avoided the need for additional imaging such as CT or radiographs, thereby minimizing unnecessary radiation exposure and aligning with the principle of "as low as reasonably achievable" (ALARA). Additionally, the inherent advantage of POCUS lies in its non-ionizing nature and immediate availability, contributing significantly to reducing healthcare costs for the patient [6-7]. Additionally, POCUS minimized delays associated with scheduling advanced imaging, enabling prompt surgical intervention to address the patient's symptoms. Surgical excision is the traditional treatment for symptomatic lipomas, especially those causing functional impairment or cosmetic concerns. Complete excision minimizes the risk of recurrence [13]. In this patient, surgical removal of the lipoma resulted in significant improvement in symptoms, allowing him to resume occupational duties without discomfort. As reported in the literature, similarities were observed in a case involving a giant palmar lipoma, where both patients had limited financial resources, functional impairment, and could not afford advanced imaging modalities such as MRI [14]. In each case, ultrasound served as a crucial and cost-effective diagnostic tool.

While MRI remains the preferred modality for detailed characterization of soft tissue masses [4]. POCUS can be sufficient when ultrasound findings are typical and the clinical suspicion for malignancy is low. Thus, in resource-limited settings or for patients with financial constraints, POCUS offers a viable and affordable diagnostic option [15]. Nonetheless, advanced imaging modalities remain indispensable for comprehensive evaluations when indicated, ensuring optimal patient outcomes through a collaborative approach between clinicians and radiologists.

## Conclusions

This case underscores the effectiveness of POCUS in evaluating giant lipomas and directly facilitating treatment without the need for additional imaging studies involving radiation exposure. POCUS provided rapid, cost-effective, and radiation-free diagnosis and management, enhancing patient care by preventing unnecessary imaging and associated delays. Surgical excision provided excellent outcomes without complications, significantly improving the patient's quality of life and work performance. However, advanced imaging modalities such as MRI may be necessary in scenarios where ultrasound findings are inconclusive or when malignancy remains a concern.
