# Correction: Utilizing Point-of-Care Ultrasound in the Diagnosis and Management of a Giant Hip Lipoma: A Case Report

**DOI:** 10.7759/cureus.c223

**Published:** 2025-05-14

**Authors:** Grant M Pham, Roger N Pham

**Affiliations:** 1 Family Medicine, Texas Tech University Health Sciences Center El Paso Paul L. Foster School of Medicine, El Paso, USA; 2 Emergency Medicine, Baylor College of Medicine, Houston, USA

The reference list has been corrected to replace references 4, 5, and 8. The incorrect references were included in the initial published version of the reference list due to a technical error.

